# Spinal Tuberculosis: Features and Early Predictive Factors of Poor Outcomes

**DOI:** 10.31138/mjr.34.2.220

**Published:** 2023-06-30

**Authors:** Soumaya Boussaid, Mariem M’rabet, Sonia Rekik, Samia Jammali, Safa Rahmouni, Khaoula Zouaoui, Hela Sahli, Mohamed Elleuch

**Affiliations:** 1 Rheumatology Department, Rabta Hospital, Tunis, Tunisia,; 2 Faculty of Medicine of Tunis, University Tunis el Manar, Tunis, Tunisia,; 3 Research unit LR 05 SP 01, La Rabta hospital

**Keywords:** spinal tuberculosis, tuberculosis, spondylitis, discitis

## Abstract

**Introduction:**

Tuberculosis is still endemic in Tunisia. Among musculoskeletal involvement, spinal tuberculosis (STB) or «Pott’s Disease» is the most common and can lead to serious neurological complications. The purpose of our study was to focus on STB features (clinical, biological, and radiological) and to identify factors associated with early unfavorable outcomes.

**Methods:**

This was a monocentric retrospective study, over a period of 20 years (2000–2020). Only patients treated appropriately were included. Patients’ informations were noted. We defined the favorable outcome criterion as weight gain, apyrexia, improvement of the general state, relief of pain, improvement in the classic inflammatory markers (CRP), and absence of vertebral deformities, neurological impairment, or sepsis. The outcome was considered unfavorable otherwise.

**Results:**

Our study involved 52 patients. Their average age was 55.21 years±17.79. The average symptom duration was 8.9 months±6.54. Spinal pain was the most common functional sign (90.4%) often inflammatory. Physical signs were dominated by segmental spinal stiffness (71.2%). Spinal magnetic resonance imaging was performed in 38 patients. The disco-vertebral biopsy puncture confirmed the diagnosis in 15 cases. All patients received anti-tuberculosis treatments with an average duration of 10.02±1.97months. The outcome at one month of follow-up was favorable in 32 cases. Poor prognosis factors were normochromic normocytic anaemia (p=0.018), initial lymphocytosis (p=0.048), and fever (p=0.01). However, vertebral fracture at standard X-ray was predictive of favorable outcome (p=0.001).

**Conclusion:**

STB is a frequent condition that needs to be treated rapidly. Poor prognosis factors were identified in this study such as normocytic normochromic anemia, initial lymphocytosis, and fever at baseline.

## INTRODUCTION

Tuberculosis (TB) is an infectious disease that experienced renewed interest in developed countries with the emergence of acquired human immunodeficiency disease (HIV)^1^ and the use of new targeted immunological therapies (biologic drugs); hence the increase in the incidence of tuberculosis in recent years.^2^. Indeed, tuberculosis was the leading cause of death from a single infectious agent, until the coronavirus pandemic (COVID-19), which is higher than HIV/AIDS. (http://www.who.int/tb/publications/global_report/en/). Although pulmonary localization is more common, other localizations, including musculoskeletal involvement, are increasing. Thus, musculoskeletal sites represent 1–2% of total TB^1^ and 10–15% of extrapulmonary TB.^1^ Spinal involvement or Pott’s disease is the most common (50% of musculoskeletal sites).^3^ The incidence of Spinal TB (STB) varies across the countries; it was found to significantly decrease over time in the US,^4^ and appears to be stable or declining in most European countries, but with an increasing proportion of cases in the foreign-born population.^5^ Recovery from this disease remains the most frequent outcome.^6^ But the prognosis is dependent on neurological, orthopaedic, or thromboembolic complications that may occur. Consequently, appropriate and early treatment is needed.^6^ Therefore, patients at risk of delay in the improvement and thus unfavorable outcomes must be identified at the early stages of the disease management. So, these patients need closer monitoring and we have to be more aggressive in their treatment to specifically avoid complications.

TB is still endemic in Tunisia with a recorded incidence of 38 per 100,000 inhabitants in 2017.^2^ Considering the burden of STB, there is a lack of studies that attempt to identify factors that impact the disease outcome; and prognostic factors have not been consistently reported.^7,8^ According to the Tunisian recommendations and those of the WHO: normalization of the thermal curve and a disappearance of nocturnal pain after a period of seven days with a gradual decrease in daily pain over a month should be noted during the management of TB.^6^ The present study, aimed to investigate STB features (clinical, biological, and radiological) as a primary endpoint. We also aimed to identify factors associated with early unfavorable outcomes (after one month of treatment) as a secondary endpoint.

## METHODS

We conducted a retrospective and descriptive study of 52 cases of STB, in the rheumatology department of La Rabta hospital in Tunis, over a period of 20 years [2000–2020]. Only patients whose clinicians determined that they had been treated appropriately, were included. The patients were treated in accordance with the guidelines for TB in Tunisia^9^ and the World Health Organization (WHO) guidelines (https://www.who.int/publications/i/item/9789240007048). Patients were excluded if they did not comply with the treatment guidelines and if the data were incomplete or the patient was lost to follow-up or had died due to other causes while receiving treatment. Patient information were noted, including i)demographic details, ii) clinical characteristics, iii) laboratory data (C-Reactive Protein (CRP), Complete Blood Count (CBC), Erythrocyte Sedimentation Rate (ESR), Sputum ± Urine BK search, and Results of skin reaction to tuberculin (SKIN TEST)), iii) imaging findings (on plain X-ray, spinal computed tomography (CT), or magnetic resonance imaging (MRI)), iiii) Bacteriological abnormalities or histological ones on CT-guided biopsy of the discovertebral site, or a biopsy of an abscess. The diagnosis of STB was based on one of the following criteria (according to the Tunisian recommendations^9^): ***i)*** either a discover-tebral biopsy puncture showing an epithelioid and giant cellular granuloma with caseous necrosis or presence of Mycobacterium tuberculosis, ***ii)*** or an association of highly suggestive epidemiological (history of tuberculous contagion, concomitant pulmonary tuberculosis), clinical (presence of abscess, strong tuberculin skin reaction, neurological signs), and radiological (very evocative image according to the Tunisian recommendations^9^) arguments. ***iii)*** or the presence of concomitant tuberculosis, or by abscess biopsy confirming the presence of Mycobacterium tuberculosis.

The treatment modalities and outcomes were also noted. Patients were assessed at Week (W)1, W2, W3, W4, at 3 months, and at the end of the treatment, according to the Tunisian guide recommendations for the management of STB.^9^

We defined the favorable outcome criterion at one month of treatment: as weight gain, apyrexia, improvement of the general state, relief of pain, improvement in the classic inflammatory markers (CRP), and absence of vertebral deformities, neurological impairment, or sepsis. The outcome was considered unfavorable otherwise.

### Statistical analysis

Data were analyzed using IBM SPSS Statistics version 26 software. We calculated simple frequencies and relative frequencies (percentages) for qualitative variables. We calculated means, medians, and standard deviations, and determined extreme values for quantitative variables. The comparisons of 2 means on independent series were carried out using Student’s t-test for independent series. The comparisons of percentages on independent series were carried out by the Pearson chi-square test, and in the event of invalidity of this test, and comparison of 2 percentages, by the two-sided Fisher exact test. In all statistical tests, the significance level was set at 0.05. The search for risk factors was carried out by calculating the Odds ratio, which represents the number of times by which the probability (risk) of an event is multiplied in the event of exposure to a factor compared to non-exposed. For the calculation of the Odds ratio, we transformed the quantitative variables into qualitative variables with two modalities. To determine the “cutoff” value, for the quantitative variable we have established ROC (Receiver Operating Characteristics) curves.

## RESULTS

### Epidemiological characteristics

Fifty-two patients, with a mean age of 55.21±17.79 years^19–91^ were included in this study. The sex ratio F/M was 2.56. The demographic, clinical, and biological characteristics of our patients are presented in **Table 1**. The mean time of symptoms duration was 8.9 months±6.54.^1–48^ The lumbar spine was the major involved site (30 patients: 65.5%) and the dorsal spine was involved in 11.5% of cases. Only five patients (9.6%) had a concomitant associated extra-vertebral localization. Concomitant pulmonary tuberculosis was diagnosed in two patients, pleural involvement was present in two other cases and one patient had associated skin localization. Radiographic abnormalities, on the X-ray of the spine, were observed in 47 patients (90.4% of cases), namely: disc narrowing (82.7%), erosion of the vertebral endplates (65.4%), and vertebral fracture (67.3%). Spinal computed tomography-scan CT-scan was performed in 28 patients (53.8%) showing: disc narrowing (44.2%), vertebral fracture (40.4%), erosion of the vertebral endplates (40.4%) and mirror geode (11.5%). Magnetic resonance imaging (MRI) was performed in 38 cases (73.1%). It was abnormal in all cases (**Table 2**).

**Table 1.** Epidemiological and clinical features of tuberculous spondylitis.

**Parameters**	**N**	**Percentage (%)**
**Epidemiological data**		
Women	37	71,2
patients >65 years	16	30,8
History of tuberculosis	5	9,6
BCG vaccination	38	73,1
contact with sick subject	6	11,5
Diabetes	8	15,4
Other immunosuppressive conditions	5	9,6
Corticosteroid therapy	4	7,7
Trauma	6	11,5
**Clinical parameters**		
Progressive setting mode	47	90,4
**Complaints**		
None (fortuitous discovery)	2	3,8
spinal pain	13	25
Spinal pain + general signs	24	46,2
Spinal pain + radiculalgia	7	13,5
Radiculalgia	5	9,6
**Diagnosis delay (late in diagnosis)**	41	78,8
**Functional signs**		
**Spinal pain**	**47**	**90,4**
lumbar	30	65,4
Inflammatory	21	40,4
**General signs**	**37**	**71,2**
Impairment of general condition	20	38,5
Fever	19	36,5
Night sweats	18	34,6
Weight loss	18	34,6
**Neurological signs**	**9**	**17,3**
Hypoesthesia	1	1,9
OTR abnormality	6	11,5
Motor deficit	1	1,9
Hypoesthesia + motor deficit	1	1,9

n: number of patients; BCG: Bacille Calmette-Guérin; OTR: Osteotendinous reflex

**Table 2.** Biological, radiological and histological features of tuberculous spondylitis.

**Parameters**	**n**	**Percentage (%)**
**Biology**		
Increase in CRP and/or ESR	38	73,1
Normochromatic normocytic anemia	28	53,8
Leukocytosis	6	11,5
Leukopenia	4	7,7
**MRI abnormalities**		
Disc pinch	34	65,4
Vertebral fracture	33	63,5
Posterior wall involvement	5	9,6
Spondylitis	19	36,5
Epiduritis	28	53,8
Signs of spinal cord compression	6	11,5
Thickness of pre-spinal soft tissues	28	53,8
Abscess or collection	33	63,4
Diffuse T2 hypersignal	30	56,5
Diffuse or circumferential signal increase in sp T1 Gado	27	50,9
Moderate and extensive condensation	32	60,4
Macrogeodes	32	60,4
Heterogeneous signal increase in sp T1	28	52,8
**DVBP**		
Epithelioid granuloma + caseous necrosis	15	28,8
Granuloma without necrosis	4	7,7
Non-specific inflammation	10	19,2
Inconclusive	9	17,3
Total	38	100
**Diagnosis arguments**		
PBDV	15	28,8
Presence of concomitant TBC	5	9,5
Highly suggestive clinical, paraclinical and scalable arguments	37	71,2

n: number of patients; MRI: Magnetic resonance imaging; Gado: Gadolinium; DVBP: Discovertebral biopsy puncture; TBC: Tuberculosis. CRP: C reactive Protein; ESR: Erythrocyte Sedimentation Rate

Only forty-three Tuberculin Skin tests (82.7%) were mentioned on patient records; twenty-seven of which (51.9%) were positives. A chest X-ray was performed in 43 cases (82.7%) of which 20 (38.5%) were normal. BK research was carried out on sputum and urine in 46 patients. Out of 46 sputum tests, three (5.8%) were positive. Of the 28 urinary tests, only one result (1.9%) was positive. A CT-guided biopsy was conducted in 41 patients (78.8%). The diagnosis of spinal tuberculosis was retained in 15 cases in front of the presence of an epithelioid and giant cellular granuloma with caseous necrosis in the results of the discovertebral biopsy, in 37 cases in front of a host of highly suggestive clinical, paraclinical and evolutionary arguments, and in 5 cases in front of the presence of associated concomitant tuberculosis (**Table 2**). An abscess biopsy was carried out in eight patients (15.4%). Two cultures (3.8%) were positive for Mycobacterium tuberculosis.

All patients received TB treatment with a total mean duration of 10.02±1.97 months [9–17]. A first-line therapy, based on HRZE, was initiated for all patients for an average time of 2.06±0.37 months.^1–3^ The transition to dual therapy, based on HR, was conducted in our patients with an average duration of 7.98±1.99 months.^6–15^

After three months of follow-up, only 50% of patients had a favorable clinical outcome and 21.2% had a CRP decrease. While at one month of follow-up, the improvement was 61.5%. This decline was mainly due to the occurrence of mechanical back pain in some patients (**Table 3**). The different complications encountered by our patients are shown in **Table 4**. Factors that have been identified as being predictive of unfavorable outcome at one month of follow-up were: normochromic normocytic anemia (p=0.018; OR=6.66), initial lymphocytosis (p=0.048), and fever (p= 0.01, OR= 9.6), (**Tables 5 and 6**). However, vertebral fracture on plain x-ray was predictive of favorable outcome (p= 0.001; OR=13). Otherwise, we found no influence of other clinicobiological and radiographic parameters on the evolutionary aspect of the disease.

**Table 3.** Post-treatment evolution of tuberculosis in W1, W2, W4, and M3.

	**Evolution at W1**		**Evolution at W2**		**Evolution at W4**		**Evolution at M3**	
	**n**	**Percentage (%)**	**n**	**Percentage (%)**	**n**	**Percentage (%)**	**n**	**Percentage (%)**
**Clinical improvement**	29	55,8	35	67,3	32	61,5	26	50
**CRP decrease**	8	15,4	9	17,3	11	21,2	11	21,2

W1: 1st week of tuberculous treatment; W2: 2nd week of tuberculous treatment; W4: 4th week of tuberculous treatment; M3: 3rd month of tuberculous treatment; n: number of patients; CRP: C-Reactive protein

**Table 4.** Distribution of Patients by complications.

**Complications**	**n**	**Percentage (%)**
**Related to the disease:**		
Spinal cord compression	3	5,8
Spinal deformity	4	7,7
Septicemia	1	1,9
**Related to anti-TBC treatment:**		
Cholestasis	3	5,8
AST and ALT increased level	2	3,8
Cholestase + AST and ALT increased level	5	9,6
Hyperuricemia	7	13,5
Major intolerance	3	5,8
**Related to bed rest:**		
thromboembolism	2	3,8
Recurrence	8	15,4

n: number of patients; anti-TBC: anti-tuberculosis; ALT: Alanine transaminase; AST: Aspartate transaminase

**Table 5.** Correlations of predisposing factors, clinical, biological, and radiological endpoints with 4-week clinical course of anti-tuberculosis therapy.

**Parameters**		**Favorable outcome**	**Unfavorable outcome**	**p**
**Age (years)**		56,09 ± 18,06	51,3 ± 16,8	0,46
**Hospitalisation duration (months)**		1,67 ± 1,18	0,92 ± 0,65	0,08
**ESR (mm/1st hour)**		70,58 ± 30,36	46,28 ± 11,42	0,054
**CRP (g/l)**		58,37 ± 61,67	46,98 ± 48,03	0,61
**Hemoglobin (g/dl)**		12,04 ± 1,06	11,04 ± 1,83	**0,03**
**VGM**		76,23 ± 17,09	87,92 ± 7,96	0,07
**CCMH**		27,57 ± 3,39	29,23 ± 3,52	0,25
**Normochromic normocytic**		10.68±0.8	9.56±0.89	**0,018**
**Anemia (g/dl)**				
**White blood cells (e/mm3)**		7365 ± 2576,19	7720 ± 2538,47	0,71
**PNN (e/mm3)**		4684,3 ± 1967,16	5150 ± 2233,66	0,67
**Lymphocytes (e/mm3)**		1782,89 ± 587,02	3360 ± 3266,48	**0,048**
**Delay in diagnosis (months)**		7,69 ± 2,75	12,7 ± 13,7	0,053
**Total duration of treatment (month)**		9,79 ± 1,61	10,38 ± 1,99	0,39
**TBC Contage**	Yes	15,62	0	
	No	84,37	100	0,19
**History of TBC**	Yes	6,25	20	0,2
	No	93,75	80	
**Diabetes**	Yes	15,62	20	
	No	84,37	80	0,75
**Corticosteroid therapy**	Yes	12,5	0	
	No	87,5	100	0,25
**Trauma**	Yes	15,62	10	
	No	84,37	90	0,66
**Spinal involvement**	Multifocal	31,25	20	
	One site	68,75	80	0,5
**Extra-vertebral TBC associated**	Yes	9,37	10	0,95
	No	90,62	90	
**Radiculalgia**	Yes	31,25	50	
	No	68,75	50	0,29
**Neurological signs**	Yes	18,75	20	
	No	81,25	80	0,93
**Impairment of general condition**	Yes	47,61	50	0,91
	No	52,38	50	
**Fever**	Yes	76,19	25	
	No	23,8	75	**0,01**
**Night sweats**	Yes	61,9	37,5	
	No	38,09	62,5	0,25
**Weight loss**	Yes	38,09	62,5	0,25
	No	61,9	37,5	
**Spinal stiffness**	Yes	71,87	70	0,91
	No	28,12	30	
**Imaging abscess**	Yes	75	70	0,76
	No	25	30	
**Standard Radio Bone Demineralisation**	Yes	36,66	22,22	0,43
	No	63,33	77,77	
**Vertebral fracture in X ray**	Yes	86,66	33,33	
	No	13,33	66,66	**0,001**
**Vertebral fracture in MRI**	Yes	83,33	70	0,26
	No	16,66	30	

p: Degree of statistical significance; ESR: Erythrocyte sedimentation rate; CRP: C Reactive Protein; VGM: Mean globular volume; CCMH: Mean corpuscular content in Hemoglobin; PNN: Polynuclear neutrophils; TBC: Tuberculosis; Values were means±SD for qualitative data and percentages for quantitative data

**Table 6.** Prognostic factors for tuberculous spondylitis.

**Prognostic Factor**	**p**	**Odds ratio**
**Normochromic normocytic anemia**	0,018	6,66
**Initial lymphocytosis**	0,048	Indefine
**Fever**	0,01	9,6
**Vertebral fracture on X ray**	0,001	13

p: Degree of statistical significance.

## DISCUSSION

Spine TB remains the most frequent form of extra-pulmonary TB and is associated with a poor quality of life in cases of delayed diagnosis and treatment.^10,11^

This study, in addition to having exposed the epidemiological, clinical, biological, and radiographic characteristics of STB, investigated the factors associated with poor early outcomes in STB. Indeed, we showed that normochromic normocytic anemia, initial lymphocytosis, and fever, are predictive of early unfavorable outcomes. However, vertebral fracture on plain x-ray is predictive of favorable outcome.

The onset age of the disease varies from one country to another, depending on the TB frequency and management strategies adopted by countries. Thus, the mean age reported is between 30 and 69.09 years.^10–14^ Accordingly, the mean age of our patients was 55.21±17.79 years and at the diagnostic time, 30.8% of our patients were older than 65 years. Young age and early diagnosis are reported as favorable prognostic factors.^15^

Several series have reported that STB is as common in women as in men.^16,17^ This may be due to the similarities in the economic, educational, and medical levels of the regions. In our study, there was a clear female predominance with a sex ratio F/M: 2.56. These data agree with some studies where a female predominance has been reported.^18–20^ However, other studies report a male predominance of STB.^21–24^

Various risk factors play an important role in the predisposition of STB; especially in Endemic regions and limited medical care areas. Among these factors: poverty, malnutrition, Immunosuppression, Diabetes mellitus, Alcohol abuse, age, and Multimorbidity, are the most common ones.^25,26^ These are also further negative predictors of the outcome.^27^ In our study; 15.4% of patients were diabetic.

The median duration of symptoms before the diagnosis is an average of six months (1–14).^28^ In our study, it was 8.9 months ± 6.54.^1–48^ This may be due to the socio-economic difficulties of patients.

STB lesions usually involve two or more vertebrae, and the most common sites are the lumbar and the thoracic spine.^17,26^ In our study, the lumbar spine was involved in 65.5% of cases and the dorsal spine was involved in 11.5% of cases. These localizations may be related to the anatomical factors with a high level of mobility and load forces in the lumbar and the thoracic region. Involvement of the longitudinal ligament may also enable multiple adjacent vertebrae involvement.^29^ The number of the affected vertebrae, greater spinal destruction (instability, deformity, defects),^26,27^ and paraplegic symptoms are also predictors of an unfavorable outcome.^30^ These data have not been proven in our study; this may be due to the small number of patients and neurological complications in our series.

Clinical manifestations of STB include systemic symptoms, weight loss, back pain, and spinal tenderness.^30^ Fatigue, night sweats, and generalized aches are found mainly in advanced stages.^31^ Fever is described only in up to 1/3 of cases.^32,33^ It was present in 36.5% of our cases. Our results suggest that it is a predictive factor of unfavorable outcomes with an OR of 9.6 (p=0.01). This needs to be confirmed by other large-scale studies.

The frequency of neurological involvement varies across studies, from 23 to 76% of patients.^34^. In our study, neurological involvement was present in 5.8% of cases. The spinal deformity was present in 7.7% of our cases. These two complications could increase the mortality in patients.^34–36^

STB is distinguished by a low systemic inflammatory activity with a moderately high Erythrocyte Sedimentation Rate and CRP. Even an augmentation of the CRP may be absent.^37^

In our study, systemic inflammatory activity was noted in 73.1% of cases, and lymphocytosis in 11.5% of cases; which is consistent with the literature.

Normochromic normocytic anemia (NNA) was present in 11.5% of our cases, it is known as a risk factor for TB.^38^ Indeed, anemia is generally a sign of malnutrition, which in itself constitutes a risk factor for tuberculosis: in a recent study of 556 patients, anemia was observed in 54.91% of them.^17^ In the same way, tuberculosis causes weight loss and malnutrition.^39^ This results in a vicious circle which can be responsible for delayed healing. Indeed, in our study, the initial NNA was correlated with an unfavorable outcome with an OR of 6.66 (p=0.018). Moreover, it was demonstrated that baseline anemia is a risk factor for persistent positive sputum smears after two months of pulmonary tuberculosis treatment.^40,41^ It has been also shown that the hazard of tuberculosis increased with anemia severity.^38^ Indeed, anemia was more frequent in the most severe clinical forms.^41^ So, some studies suggest that anemia can be considered a biomarker of TB severity.^42^ Thus, we suggest paying more attention to nutritional supplementation before, during, and after TB treatment.^17^

In addition to anemia, lymphocytosis is common in tuberculosis. In fact, the immune response to M. tuberculosis is mediated by T lymphocytes.^43^ We reported in our study that lymphocytosis was correlated with disease severity (p=0.048); that has not yet been reported in the literature, which requires more studies with a larger sample size of patients to better elucidate this correlation.

Because of the delay in diagnosis during STB, radiographic abnormalities suggestive of spondylodiscitis are often present at the start of the patient care.^44^ In accordance with these data, radiographic abnormalities were observed in 47 patients in our study. We further found that the presence of a vertebral fracture on the initial spinal X-ray was correlated with a favorable outcome. This can be explained by the presence of the peri-fracture hematoma, thus allowing better vascularization of the bone, and hence a better penetration of the antibiotic into the infectious focus.

Our study, despite its limitations (retrospective with a limited sample size), allowed us to identify a new finding of early predictive factors of the delay in the improvement. Indeed, close and regular follow-up of these patients is necessary in order to better control the disease and avoid further complications.

## CONCLUSION

Screening for early diagnosis of STB is imperative, especially in TB-endemic resource-poor countries. Based on the analysis of demographic, clinical, and biological variables, we showed through this study that normochromic normocytic anemia, initial lymphocytosis, and fever are predictive of early unfavorable outcomes. Future prospective studies in countries with diverse TB burdens are needed to confirm the prognostic factors for early unfavorable outcomes in patients with STB.

## Data Availability

The datasets used and/or analysed during the current study are available from the corresponding author on reasonable request.

## ABBREVIATIONS

CBC: Complete Blood Count
CRP: C-Reactive Protein
CT: Computed tomography
ESR: Erythrocyte Sedimentation Rate
HIV: Human Immunodeficiency Virus
MRI: Magnetic resonance imaging
ROC: Receiver Operating Characteristics
STB: Spinal tuberculosis
TB: Tuberculosis
WHO: World Health Organization
